# How to improve situation assessment and decision-making in a simulated mass casualty incident by using an unmanned aerial vehicle

**DOI:** 10.1186/1757-7241-22-S1-P2

**Published:** 2014-07-07

**Authors:** Håkon B Abrahamsen

**Affiliations:** 1 Department of Research and Development, The Norwegian Air Ambulance Foundation, Drøbak, Norway; 2 Department of Global Health and Primary Care, University of Bergen, Bergen, Norway; 3 National Centre for Emergency Primary Health Care, Uni Research, Bergen, Norway

## Background

Situation assessment and decision-making is challenging in the complex task environment of pre-hospital critical care. Human attention is a limited resource, critical information is often missing and cues in the situation may be misinterpreted, misdiagnosed or ignored. We evaluated the feasibility of using a remotely piloted unmanned aerial vehicle (UAV) to gather information from the air in order to support decision-making in a simulated major incident.

## Methods

A mass casualty incident was constructed to mimic a car against bus accident with 25 entrapped school children [1]. A radio controlled, multi rotor, electrical UAV was equipped with a daylight - and an infrared video camera and was directed into the location. Video from the site of the accident was wirelessly streamed to a ground station.

## Results

The aerial vehicle was maneuvered at different altitudes and positions in and around the area of interest with good accuracy. Video from the rescue operation was recorded using the daylight camera, and the infrared camera was able to localize people that were trapped in the dark inside the bus. Video streaming from the air provided crucial information about the course of events and status at the scene that would not have been possible to obtain from the ground. We achieved useful information on how to collect, interpret and make use of the data to improve the processes of assessing the scene, make sound decisions and distribute the recourses according to need.

## Conclusion

Rotor-wing unmanned aerial vehicles equipped with video cameras can provide useful decision making support in major incidents. They seem especially useful in gathering information in high-risk work environments, when the access to patients is limited or restricted and in mass casualties where the geographical extent of an event is large.
